# A Model to Discriminate Malignant from Benign Thyroid Nodules Using Artificial Neural Network

**DOI:** 10.1371/journal.pone.0082211

**Published:** 2013-12-16

**Authors:** Lu-Cheng Zhu, Yun-Liang Ye, Wen-Hua Luo, Meng Su, Hang-Ping Wei, Xue-Bang Zhang, Juan Wei, Chang-Lin Zou

**Affiliations:** 1 Department of Radiation Oncology and Chemotherapy, The First Affiliated Hospital of Wenzhou Medical College, Wenzhou, China; 2 Department of Oncology, The First Affiliated Hospital of Wenzhou Medical College, Wenzhou, China; Florida International University, United States of America

## Abstract

**Objective:**

This study aimed to construct a model for using in differentiating benign and malignant nodules with the artificial neural network and to increase the objective diagnostic accuracy of US.

**Materials and methods:**

618 consecutive patients (528 women, 161 men) with 689 thyroid nodules (425 malignant and 264 benign nodules) were enrolled in the present study. The presence and absence of each sonographic feature was assessed for each nodule - shape, margin, echogenicity, internal composition, presence of calcifications, peripheral halo and vascularity on color Doppler. The variables meet the following criteria: important sonographic features and statistically significant difference were selected as the input layer to build the ANN for predicting the malignancy of nodules.

**Results:**

Six sonographic features including shape (Taller than wide, p<0.001), margin (Not Well-circumscribed, p<0.001), echogenicity (Hypoechogenicity, p<0.001), internal composition (Solid, p<0.001), presence of calcifications (Microcalcification, p<0.001) and peripheral halo (Absent, p<0.001) were significantly associated with malignant nodules. A three-layer 6-8-1 feed-forward ANN model was built. In the training cohort, the accuracy of the ANN in predicting malignancy of thyroid nodules was 82.3% (AUROC = 0.818), the sensitivity and specificity was 84.5% and 79.1%, respectively. In the validation cohort, the accuracy, sensitivity and specificity was 83.1%, 83.8% and 81.8%, respectively. The AUROC was 0.828.

**Conclusion:**

ANN constructed by sonographic features can discriminate benign and malignant thyroid nodules with high diagnostic accuracy.

## Introduction

Nodular thyroid disease is a common finding in the general population, particularly in iodine-deficient areas. The prevalence of palpable nodules in population is 3% to 4%, and the prevalence of nonpalpable nodules incidentally identified on imaging approaches 40% to 50% after the age of 60 years[1]–[5]. The diagnosis of thyroid cancer relies on cervical ultrasound and fine-needle aspiration (FNA) biopsy, which collects cells for cytological examination [6], [7]. FNA cytology is currently the most reliable diagnostic tool for evaluation of thyroid nodules. It provides a definitive diagnosis of benign or malignant thyroid disease in most cases. However, in 20% to 30% of nodules, FNA cytology cannot reliably rule out cancer, and such cases are reported as indeterminate for malignancy [8], [9]. To improve the diagnosis accuracy, new diagnostic approaches combined FNA cytology and molecular biomarkers were proposed in recent years [10]–[12]. In additional, CT and MRI have a limited role in the initial evaluation of solitary nodule and their indications include suspected tracheal involvement, either by invasion or compression, extension into the mediastinum, or recurrent disease[5], [13]–[15].

Though FNA biopsy can differentiate malignant and benign nodules in most cases, it is an invasive procedure after all and uncomfortable for the patient [14], [15]. Ultrasonography (US) is a powerful imaging technique for identifying thyroid nodules, which are very common in clinical practice. It is a cost-effective, noninvasive, portable, and safe imaging modality in the evaluation for detection of nonpalpable thyroid cancers, it has barely drawbacks expect for its low sensitivity. The incidence of thyroid nodules detected by US ranges from 10% to 67% [16]–[19]. The great majority of nodules are benign, yet the clinical importance lies in the detection of malignancy, which comprises approximately 2.7–17% of all thyroid nodules. US has been widely used to distinguish benign from malignant nodules using several sonographic characteristics. However, no single ultrasound feature has the adequate diagnostic accuracy for diagnosing malignant nodules.

The artificial neural network (ANN) is a novel computer model inspired by the working of the human brain. It can build nonlinear statistical models to deal with complex biological systems. ANN models have several advantages of over statistical methods. It can rapidly recognize linear patterns, non-linear patterns with threshold impacts, categorical, step-wise linear, or even contingency effects[20]. Analyses by ANN need not start with a hypothesis or a priori identification of potentially key variables, so undocumented or quantitated potential prognostic factors can be determinate if they already exist in the masses of datasets, though they might have been overlooked in the past. It can build nonlinear statistical models to deal with complex biological systems. In recent years, ANN models have been introduced in clinical medicine for clinical validations [21]–[23]. In this study, we aimed to construct a model for using in differentiating benign and malignant nodules with the artificial neural network and to increase the objective diagnostic accuracy of US.

## Materials and Methods

### Patients

We conducted our retrospective study extend from January 2010 and December 2012. 618 consecutive patients with 689 thyroid nodules were enrolled in the present study. All the patients had undergone US and US-guided FNAB preoperatively and subsequently undertook surgery. Pathological results were used as the reference standards. The enrollment criteria for the patients were as follows: (1) there was no thyroid diseases history. (2) There was no radiation history on neck. (3) All patients underwent US and FNAB examinations. All the patients were from eastern China (most of the patients were from Wenzhou City) and received primary treatment in our hospital.

### Ethics

Written informed consent was obtained from the patient for publication of this report. The study was approved by the Ethics Committee of The First Affiliated Hospital of Wenzhou Medical College, Wenzhou, China.

### Clinicopathological and US features

Thyroid ultrasound examinations were performed by two experience technicians with an Acuson Sequoia and 128XP sonographic scanners (Siemens Medical Solutions, Mountain View, CA) equipped with commercially available 8- to 13-MHz linear probes. The following sonographic features were assessed for each nodule: shape, margin, echogenicity, internal composition, presence of calcifications, peripheral halo and vascularity on color Doppler. The shape of the nodule was classified as taller than width measured in transverse dimension or wider than tall. Margins of nodules were categorized as well circumscribed when clear demarcation with normal thyroid was noted, and as not well circumscribed, which included irregular and microlobulated margins. The echogenicity of each nodule was classified as hypo-, iso- or hyperechoic in comparison with the normal background thyroid tissue. A nodule was defined as marked hypoechoic, when a nodule was hypoechoic relative to adjacent strap muscles. The echo structure was defined as solid, cystic or predominantly cystic. Predominantly cystic nodules were those containing cystic components that constituted more than an estimated 50% of the lesion. The presence of micro- and macrocalcifications was documented. Microcalcifications were defined as tiny, punctuate echogenic foci of 1 mm or less either with or without posterior shadowing. Microcalcifications were defined as larger than 1 mm. The vascularity on color Doppler was classified as absent, present flow. The status of nodules was confirmed by a final histological examination after surgery.

### Statistical Analysis and Neural Network analysis

Statistical analysis was performed using SPSS 20.0 software (SPSS Inc., Chicago, IL, USA). Continuous variables were expressed by mean ± standard deviation and compared using student's t-test when necessary. Categorical variables were described by proportions or count and compared using proportions chi-square test or the Fisher's exact test when necessary. Univariate analysis was applied to assess the relationship between sonographic features (input variables) and malignancy (output variables). The variables we selected as the input layer to build the ANN for predicting the malignancy of nodules were required to meet the following criteria: important sonographic features and statistically significant difference.

In this study, we built an ANN by using the Matlab 8.0 (The Match Works Inc., Natick, USA) Variables found to be significantly related to the malignancy of nodules were selected to build the ANN. 689 eligible nodules were assigned to a training cohort (n = 464; 67%) and a validation cohort (n = 225; 33%) randomly using *rv.bernoulli* method. One of the major limitations of ANN is over-training, which can lead to good performance on training sets but poor performance on relatively independent validation sets. To avoid over-training during building of the ANN, 332 patients (72%) were again randomly selected from the training group to train the network and the remaining 132 (28%) were used for cross-validation. The learning mechanism applied on this ANN was BP by calculating the errors between output value and desired output value. Then, the weight of the connections was altered between neurons to decrease the overall errors of the network. Training was terminated when the sum of square errors was at minimum, compared with the cross-validation data set. The activation function, representing the outcomes of ANN, was used with continuous outputs on the interval from 0 to 1, in which 0 = benign, 1 = malignant. To avoid different inter nodules variability, we repeated the process only excluding nodules derived from the same patient and total 561 nodules were taking in the study.

## Results

### Baseline characteristic of thyroid nodules

A total of 689 thyroid nodules were enrolled in the study. The clinicopathologic data of all patients was listed in Table 1. The size of the nodules ranged from 4 mm to 52 mm (mean size 13.3 mm ±6.5). We found no statistical difference between the benign and malignant nodules with regard to size. A taller than wide shape was found more frequently in malignant nodules (56.5%) than in benign nodules (23.5%). Hypoechogenicity (including the subgroup of markedly hypoechoic nodules) was a sonographic feature to be found in a substantial number of malignant nodules (81.4%). The frequency of hypoechogenicity in benign nodules was low (50.8%). The presence of microcalcifications and intranodular vascularity on Doppler examination in malignant nodules was significantly higher than in benign ones. However, no significant difference was found on intranodular vascularity on Doppler examination between benign and malignant nodules (Table 1). Specific characteristics of the training and validation cohorts used to build and test the ANN are described in Table 2.

**Table 1:** Patients characteristics and ultrasonographic Features of Benign and Malignant Thyroid Nodules.

Characteristics	Benign Nodules	Malignant Nodules	P Value
Total Number	264	425	
**Age at diagnosis**	48.2±10.8	47.4±11.1	0.350
<45	111 (42.0)	174 (40.9)	0.775
≥45	153 (58.0)	251 (59.1)	
**Sex**			0.048
Female	213 (80.7)	315 (74.1)	
Male	51 (19.3)	110 (25.9)	
**Shape**			<0.001
Taller than wide	62 (23.5)	240 (56.5)	
Wider than tall	202 (76.5)	185 (43.5)	
**Composition**			<0.001
Solid	151 (57.2)	410 (96.5)	
Cystic or mixed	113 (42.8)	15 (3.5)	
**Echogenicity**			<0.001
Hyperechogenicity/Isoechoic	130 (49.2)	79 (18.6)	
Hypoechogenicity	134 (50.8)	346 (81.4)	
**Calcification**			<0.001
Absence	193 (73.1)	128 (30.1)	
Microcalcification	36 (13.6)	265 (62.4)	
Other calcification	35 (13.3)	32 (7.5)	
**Margin**			<0.001
Well-circumscribed	236 (89.4)	278 (65.4)	
Not Well-circumscribed	28 (10.6)	147 (34.6)	
**Vascularity**			0.469
Absent	233 (88.3)	367 (86.4)	
Present	31 (11.7)	58 (13.6)	
**Peripheral Halo**			<0.001
Absent	5 (1.9)	137 (32.2)	
Present	259 (98.1)	288 (67.8)	

**Table 2:** Baseline characteristics of the study nodules stratified by ANN cohorts.

Characteristics	Training Group	Validation Group	P Value
Total Number	464	225	
**Shape** (Taller than wide)	205 (44.2)	97 (43.1)	0.791
**Composition** (Solid)	378 (81.5)	183 (81.3)	0.967
**Echogenicity** (Hypoechogenicity)	322 (69.4)	158 (70.2)	0.825
**Calcification**			0.484
Microcalcification	196 (42.2)	105 (46.7)	
Other calcification	48 (10.3)	19 (8.4)	
**Margin** (Not Well-circumscribed)	123 (26.5)	52 (23.1)	0.337
**Peripheral Halo** (Present)	365 (78.7)	182 (80.9)	0.498

### Construction of ANN

As shown in Table 1, the sonographic features including shape (Taller than wide, p<0.001), margin (Not Well-circumscribed, p<0.001), echogenicity (Hypoechogenicity, p<0.001), internal composition (Solid, p<0.001), presence of calcifications (Microcalcification, p<0.001) and peripheral halo (Absent, p<0.001) were significantly associated with malignant nodules at the Univariate analysis which were all used to build the ANN.

**Figure 1:**
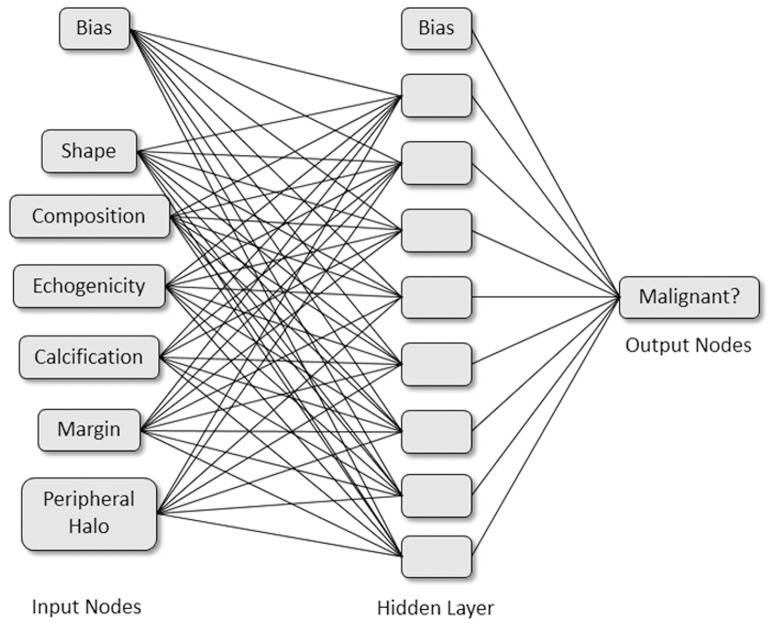
Schematic representation of the artificial neural network developed to distinguish malignancy of thyroid nodules.

Multilayer perceptron (MLP) is one of the most popular and mature ANN architectures with a feed forward neural network where processing neurons are grouped into layers and connected by weighted links. We therefore established an ANN model using MLP. In this present study, MLP included the input, hidden and output layers. Neurons were linked with weighted connections (Fig. 1). In general, the number of input variables and output variables were respectively equal to the number of sonographic features and malignancy of thyroid nodules we set. As Fig. 1 shows, the MLP has six input neurons and one output neuron. After the debugging and testing five times, eight hidden neurons were added to the hidden layer to increase the MLP's performance.

### Assessment of the predictive accuracy of ANN

**Figure 2:**
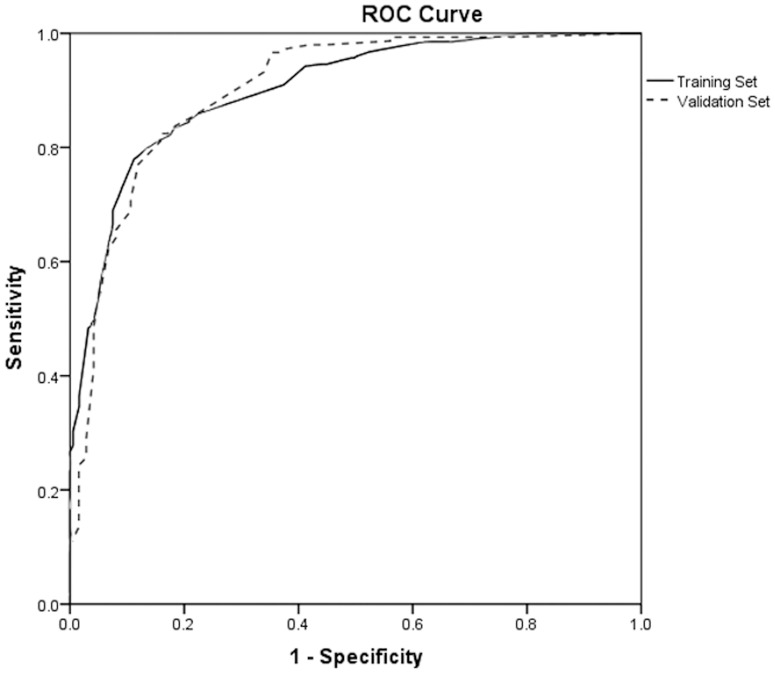
Receiver operating characteristic curve analysis of the predictive accuracy of the models to predict malignancy of thyroid nodules in the training and validation cohorts.

In the training cohort, the accuracy of the ANN in predicting malignancy of thyroid nodules was 82.3% (AUROC = 0.818, 95% CI: 0.780–0.852, p<0.001), the sensitivity, specificity malignancy predictive value (positive predictive value, PPV) and benignity predictive value (negative predictive value, NPV) was 84.5%, 79.1%, 85.7% and 77.5%, respectively (Table 3, Fig. 2). When the ANN was finally evaluated in the validation cohort, the accuracy, sensitivity, specificity, PPV and NPV was 83.1%, 83.8%, 81.8%, 89.9% and 72.4%, respectively (Table 3). The AUROC was 0.828, 95% CI: 0.772–0.875, p<0.001 (Fig. 2). When excluding nodules derived from the same patient, total 561 nodules were enrolled in study, we obtained a similar results (Table 4).

**Table 3:** Classification Accuracy of ANN in Training and Validation Groups (689 nodules).

Group by ANN model	Group by pathology in training set		Group by pathology in validation set	
	Benign	Malignant	Benign	Malignant
Benign	148	43	63	24
Malignant	39	234	14	124
Total	187	277	77	148
Sensitivity		84.5% (234/277)		83.8% (124/148)
Specificity		79.1% (148/187)		81.8% (63/77)
Accuracy		82.3% (382/464)		83.1% (187/225)
PPV		85.7% (234/273)		89.9% (124/138)
NPV		77.5% (148/191)		72.4% (63/87)

*ANN* artificial neural network; *PPV* positive predictive value; *NPV* negative predictive value.

**Table 4:** Classification Accuracy of ANN in Training and Validation Groups (561 nodules).

Group by ANN model	Group by pathology in training set		Group by pathology in validation set	
	Benign	Malignant	Benign	Malignant
Benign	97	33	39	20
Malignant	26	216	9	121
Total	123	249	48	141
Sensitivity		86.7% (216/249)		85.8% (121/141)
Specificity		78.9% (97/123)		81.3% (39/48)
Accuracy		84.1% (313/372)		84.7% (160/189)
PPV		89.3% (216/242)		93.1% (121/130)
NPV		74.6% (97/130)		66.1% (39/59)

*ANN* artificial neural network; *PPV* positive predictive value; *NPV* negative predictive value.

## Discussion

It is well known that none of the single sonographic features allows to differentiate malignant from benign thyroid lesions. However, finding in US image of nodule one or more than one suspicious features, correlates well with the risk of malignancy[19]. In our study, we found that six sonographic features, including shape, margin, echogenicity, internal composition, presence of calcifications and peripheral halo, could be used for the discrimination of the thyroid nodules.

In the study of Kim et al [24], suspicious sonographic features were defined as irregular or microlobulated margin, marked hypoechogenicity, microcalcifications and a shape that was more tall than it was wide. In the presence of even one of these sonographic findings the sensitivity, specificity and accuracy were 93.8%, 66% and 74.8%, respectively. Moon et al [25] evaluated the diagnostic accuracy of US for the depiction of benign and malignant thyroid nodules and found that the US criteria including a shape taller than wide, a spiculated margin, marked hypoechogenicity, microcalcification and macrocalcification were helpful for discrimination of malignant nodules from benign ones. According to their results, the diagnostic accuracy for the nodules one centimeter or less in size was 77% when one of the five malignant findings was used. Other studies [26]–[28] found the same sonographic features.

Color Doppler sonography can aid in the prediction of thyroid malignancy. Internal flow is suggestive of malignancy [29], [30], but this technique cannot be used to exclude malignancy. According to a previous study[30], 14% of solid non-hypervascular nodules were malignant. In our study, there was no difference found in vascularity between benign and malignant nodules. Therefore, Color Doppler imaging was not used in our study.

Many authors [31]–[34] reported that the combination of ultrasound features makes the diagnosis of a malignant nodule more probable. In these previous study, each suspicious US feature was summed as the same weight, even though each US feature has a different probability of malignancy. Therefore, the risk of malignancy was higher in a thyroid nodule with one suspicious US feature, such as a microcalcification or microlobulated margin than for a thyroid module with 2 suspicious malignant US features (solid composition and hypoechogenicity). Kwak et al [35] developed a model that the suspicious US feature had a different risk score according to their ORs in thyroid malignancy. However, most of the sonographic features have multidimensional and nonlinear relationship. So it is ideally difficult to predict the malignancy with a conventional statistical technique. Neural networks offer a number of advantages, including requiring less formal statistical training, ability to implicitly detect complex nonlinear relationships between dependent and independent variables, ability to detect all possible interactions between predictor variables, and the availability of multiple training algorithms. However, ANN also requires large amounts of training data and there was no uniform standard in choosing network structure.

In the present study, six sonographic features including shape, margin, echogenicity, internal composition, presence of calcifications and peripheral halo were significantly associated with malignant nodules. Then we built a three-layer 6-8-1 feed-forward ANN model including these six sonographic features as input neurons. In the training cohort, the accuracy of the ANN in predicting malignancy of thyroid nodules was 82.3% (AUROC = 0.818), the sensitivity and specificity was 84.5% and 79.1%, respectively. In the validation cohort, the accuracy, sensitivity and specificity was 83.1%, 83.8% and 81.8%, respectively. The AUROC was 0.828.

In **conclusion**, ANN constructed by sonographic features can discriminate benign and malignant thyroid nodules with high diagnostic accuracy.
